# Congenitally blind adults can learn to identify face-shapes via auditory sensory substitution and successfully generalize some of the learned features

**DOI:** 10.1038/s41598-022-08187-z

**Published:** 2022-03-14

**Authors:** Roni Arbel, Benedetta Heimler, Amir Amedi

**Affiliations:** 1 Department of Medical Neurobiology, Hebrew University of Jerusalem, Hadassah Ein-Kerem, Jerusalem, Israel; 2 The Institute for Brain, Mind and Technology, Ivcher School of Psychology, Reichman University, Herzeliya, Israel; 3 Department of Pediatrics, Hadassah Mount Scopus Hospital, Jerusalem, Israel; 4 Center of Advanced Technologies in Rehabilitation (CATR), The Chaim Sheba Medical Center, Tel Hashomer, Israel

**Keywords:** Perception, Human behaviour, Learning and memory, Sensory processing

## Abstract

Unlike sighted individuals, congenitally blind individuals have little to no experience with face shapes. Instead, they rely on non-shape cues, such as voices, to perform character identification. The extent to which face-shape perception can be learned in adulthood via a different sensory modality (i.e., not vision) remains poorly explored. We used a visual-to-auditory Sensory Substitution Device (SSD) that enables conversion of visual images to the auditory modality while preserving their visual characteristics. Expert SSD users were systematically taught to identify cartoon faces via audition. Following a tailored training program lasting ~ 12 h, congenitally blind participants successfully identified six trained faces with high accuracy. Furthermore, they effectively generalized their identification to the untrained, inverted orientation of the learned faces. Finally, after completing the extensive 12-h training program, participants learned six new faces within 2 additional hours of training, suggesting internalization of face-identification processes. Our results document for the first time that facial features can be processed through audition, even in the absence of visual experience across the lifespan. Overall, these findings have important implications for both non-visual object recognition and visual rehabilitation practices and prompt the study of the neural processes underlying auditory face perception in the absence of vision.

## Introduction

Faces represent a common category of visual objects, and identification of their exemplars is the most prevalent object-identification task performed by sighted individuals^1^. Among other visual categories of objects, including man-made objects, words, and numbers, faces are unique, as their identification is performed almost exclusively through the visual modality. For example, man-made objects are easily accessible and therefore also identifiable via tactile manipulation in sighted and in blind individuals^2^, and reading is accessible via the tactile modality using the Braille code^3^. Another factor separating faces from other categories of visual objects is that expertise in face identification continues to evolve through the first decade of life^4,5^, with evidence of different processes mediating face identification in different developmental stages and clinical conditions. Specifically, in young children and those with face-identification impairments, face identification is based on featural processing, with a gradual development of adult-like holistic face processing at around school age^6–8^. This change in the perceptual process underlying face perception is accompanied by the development of a neural network for face processing, including regions in the visual cortex presenting cortical selectivity to faces over other visual stimuli^4^.

In sum, while sighted individuals consistently engage the vast cognitive, perceptual, and neural resources required to perform face identification, congenitally blind individuals represent a unique group, excluded from the common experience of face-shapes since birth.

This raises the question of whether accurate identification of different face exemplars based only on their shape-features can arise in blind people. Indeed, although the blind population can, in principle, perceive face shapes via touch, tactile face exploration has several crucial limitations. First, it does not happen very often, mainly due to social conventions. Consequently, congenitally blind adults are exposed to facial shapes less than their sighted peers. Thus, through tactile exploration blind people might learn the main perceptual characteristics of faces, but they might develop a limited ability to differentiate among face exemplars—if any at all. Indeed, face-shapes are a quite homogenous category of objects and humans need extensive exposure to a lot of different exemplars to reach an accurate face-discrimination ability. Second, unlike visual perception, during tactile perception each face part is explored separately, therefore further limiting the ability to construct holistic face representations^9^ which might facilitate identification processes. Both of the aforementioned reasons may therefore prevent the mental creation of a complete face representation in congenitally blind adults, potentially limiting face-shape perception in this population^9^.

Here we investigate for the first time the extent to which congenitally blind people can learn to perceive, identify and therefore accurately discriminate among face-shapes during adulthood via an atypical sensory modality. To this end, we transformed visual faces into audition, using an in-built visual-to-auditory transformation algorithm (i.e., sensory substitution). We chose audition because it is a sensory modality offering relatively higher resolution in frequency and time than touch^10^, therefore potentially allowing to convey richer face-related information. In addition, audition is not typically used for shape-based object recognition, including face-shapes, in everyday life, therefore excluding that resulting learning may be facilitated by previous experience and training. Specifically, visual-to-auditory Sensory Substitution Devices (SSDs) translate information carried via vision into audition using in-built algorithms that preserve the exact shape and location of features, creating what we call soundscapes, the interpretation of which can be learned by users via specific training^11,12^. Previous studies conducted on congenitally blind adults demonstrated the efficacy of visual-to-auditory SSD for various object-recognition tasks. Yet because of the need to down-sample each image to optimize SSD transformations, and given the lack of SSD training programs for advanced users, previous SSD studies on auditory shape recognition focused on simple images^11,13^. Full faces were excluded because these complex visual images contain many details that must be correctly perceived to allow identification of specific exemplars, making it an extremely difficult SSD training—if possible at all. With the current study, we therefore also aimed to test the limits of SSD learning via audition, i.e., the extent to which it is possible to learn to perceive complex visual objects through sound, ultimately also starting to address the open issue regarding the extent to which current SSDs can be used as rehabilitation tools for everyday life conditions^14^. We reasoned that our in-built visual-to-auditory SSD algorithm, called the EyeMusic^11,12^ might facilitate the learning of complex soundscapes due to the fact that it embeds the transformation into sound of a unique visual dimension lacking in other visual-to-auditory SSDs, namely, color (see below and see “Methods” for details on the algorithm’s transformation rules). Indeed, color is known to enhance discriminability of images, especially in lower resolutions, and has been directly shown to enhance real-life face identification in the context of low visual resolution^15^. While it is difficult to directly project on the blind population the same color-related benefits, we reasoned that the EyeMusic color-transformation may facilitate the differentiation among auditory face features, i.e., similarly to what color does for hard visual tasks (see also^16^ showing the benefits of adding the EyeMusic color feature to the reading of words composed of letters from a newly learned SSD alphabet).

In greater detail, the EyeMusic is based on the sweep-line technique in which an image is scanned from left to right; each pixel of the image is translated into a musical note based on its location in the image, as well as its color. Specifically, pixels located lower on the image are translated into sounds of a lower musical note, while pixels located high on the image are translated into sounds of a higher musical note. In addition, positioning in the x-axis is conveyed via time, such that portions of the image located more to the left will be heard first. Five colors are represented by five musical instruments (red is mapped to an organ note, green to a reed’s tones, blue to brass instruments, yellow to string instruments, and white to voices of a choir; black is represented by silence).

We developed a specific methodology to train a group of congenitally blind adults, who have had already 50–70 h of past experience with SSDs, to identify complicated multi-colored images of cartoon faces. First, to engage participants in the challenging training program, lasting ~ 12 h, and to promote efficient learning^17,18^, we inserted the training within the ecological situation of the “Guess Who?” children’s game. Participants were familiar with the game, yet until now had no accessible way to participate in. Notably, despite their previous SSD experience, soundscapes of face-shapes were for our participants a behavioral jump compared to previously trained soundscapes, as briefly stated also above. Indeed, face-shape soundscapes included multi-colored filled visual images that when transformed into audition, required the analysis of numerous sounds simultaneously (see^19^ for a discussion of the challenges in discriminating features on the Y axis in sweep-line based SSDs). Second, in order to facilitate and simplify the learning of these complex face-shape soundscapes and to allow participants to familiarize with the resulting complex sounds, we developed the strip-method to be used during the early stages of the training program, i.e., before introducing the full soundscapes. Specifically, we divided each visual face in three horizontal strips (upper, middle and lower face parts) and presented to participants one strip at a time (see “Methods” for further details and see Fig. 2 and “Supplementary material” for all our soundscapes). At the end of the program, each participant was tested on several tasks including face-shape identification. In addition, we tested whether face-shape identification could extend to the (untrained) inverted orientation of the same faces. We reasoned this was a particularly hard task as the inverted images created entirely different soundscapes from those of the trained faces. In addition, to perform identification of the inverted faces, participants were supposed to manipulate the inverted stimulus (e.g., mentally rotate it) to perform a correct face identification. Therefore, with this task, we aimed at testing the extent to which our participants were able to manipulate the learned face properties. Lastly, we tested face-learning dynamics, asking whether the principles learned during the ~ 12-h intense training program could be quickly applied to a novel set of cartoon faces. To this aim, we trained participants to perceive and learn to identify a novel set of 6 cartoon faces in 2 h of additional training, and compared this rapid learning to the learning of the first cohort of faces.

## Results

### Part 1: Interim exploratory testing of face training progress (after 6 h of training)

#### *Assessment 1: guess who game: yes/no questions on facial features and naming task (n* = *7)*

**Figure 1:**
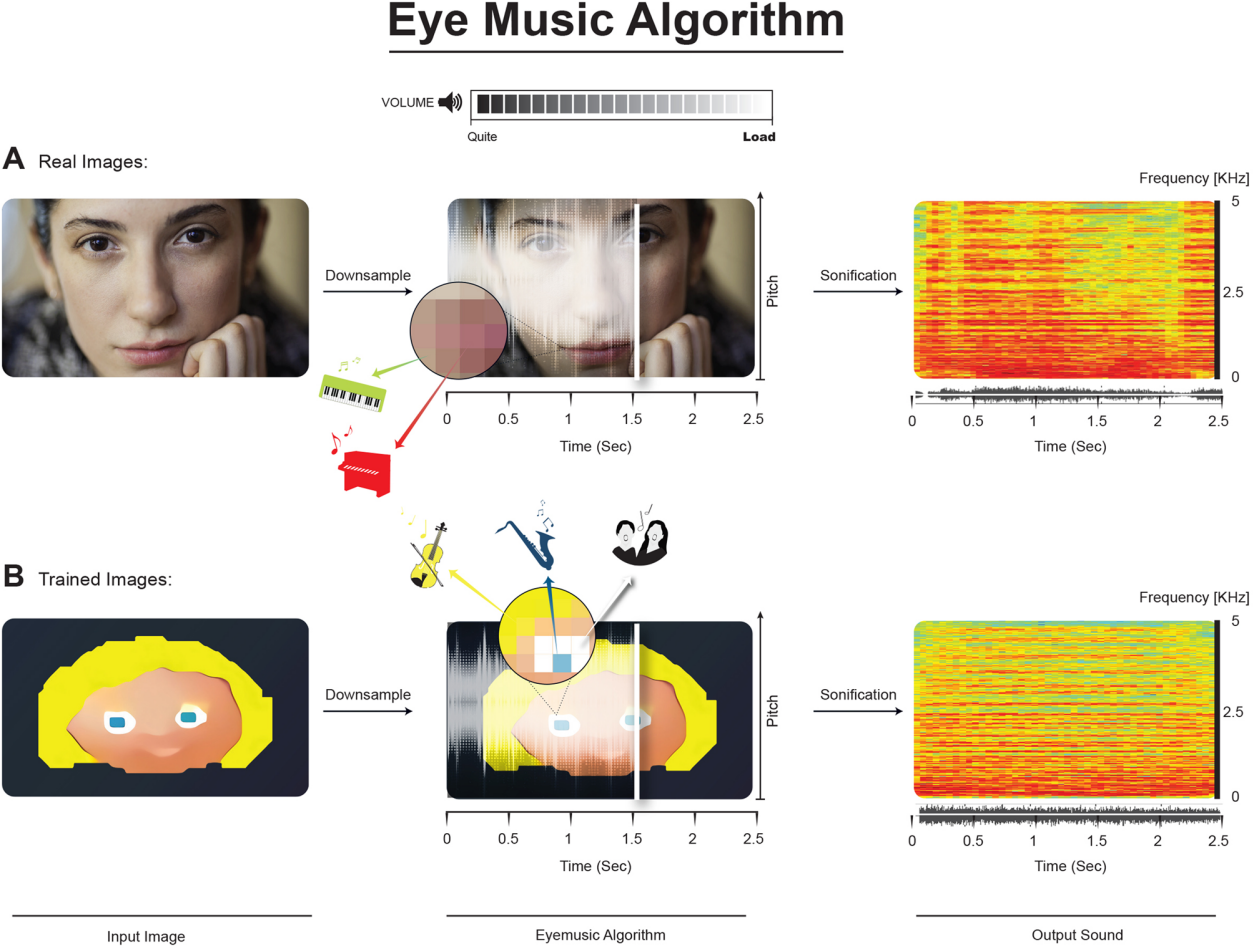
Training congenitally blind adults’ auditory identification of cartoon faces. Eye-Music transformation algorithm: Each image is scanned from left-to-right using a sweep-line approach so that the x-axis is mapped to time (i.e., characters positioned more on the left of the image are heard first). After downsampling the image to the EyeMusic’s resolution (50 × 30 pixels) the y-axis is mapped to the frequency domain using the pentatonic scale, such that parts of a character which appear higher in the image will be sonified with a higher pitch. Color is mapped to musical instruments. Red, white, blue, yellow, and green are transformed into organ notes, choir, brass instruments, string instruments, and reed tones, respectively. (**A**) An example of a real face transformed via the EyeMusic algorithm. (**B**) An example of a trained (cartoon) image transformed via the EyeMusic algorithm.

To explore participants ability in extracting shape-features among the soundscapes following the first stage of face-training, we asked them yes/no questions regarding the faces, and registered their verbal responses. Questions were directed towards face-features, such as “does this character wear glasses?”, “is this character bearded?”. Results showed participants correctly answered questions with 72.77% accuracy (standard deviation—SD =  ± 5.33%, chance level 50%) (Fig. 3A). When separating face features into visually large details (such as hair, beard, etc.) and visually small details (glasses, eyes, etc.), participants were able to identify large visual features with 93.41% accuracy (SD =  ± 5.31%) and small features with 58.65% accuracy (SD =  ± 8.28%) (Fig. 1A).

After responding to all feature-related questions for each face, participants were asked to name the face (once per face in this first assessment). Participants were able to name faces with 66.67% accuracy (SD =  ± 21.52%, chance level 16.7% Fig. 1A).

#### *Assessment 2: Detection of change in facial features (n* = *7)*

**Figure 2:**
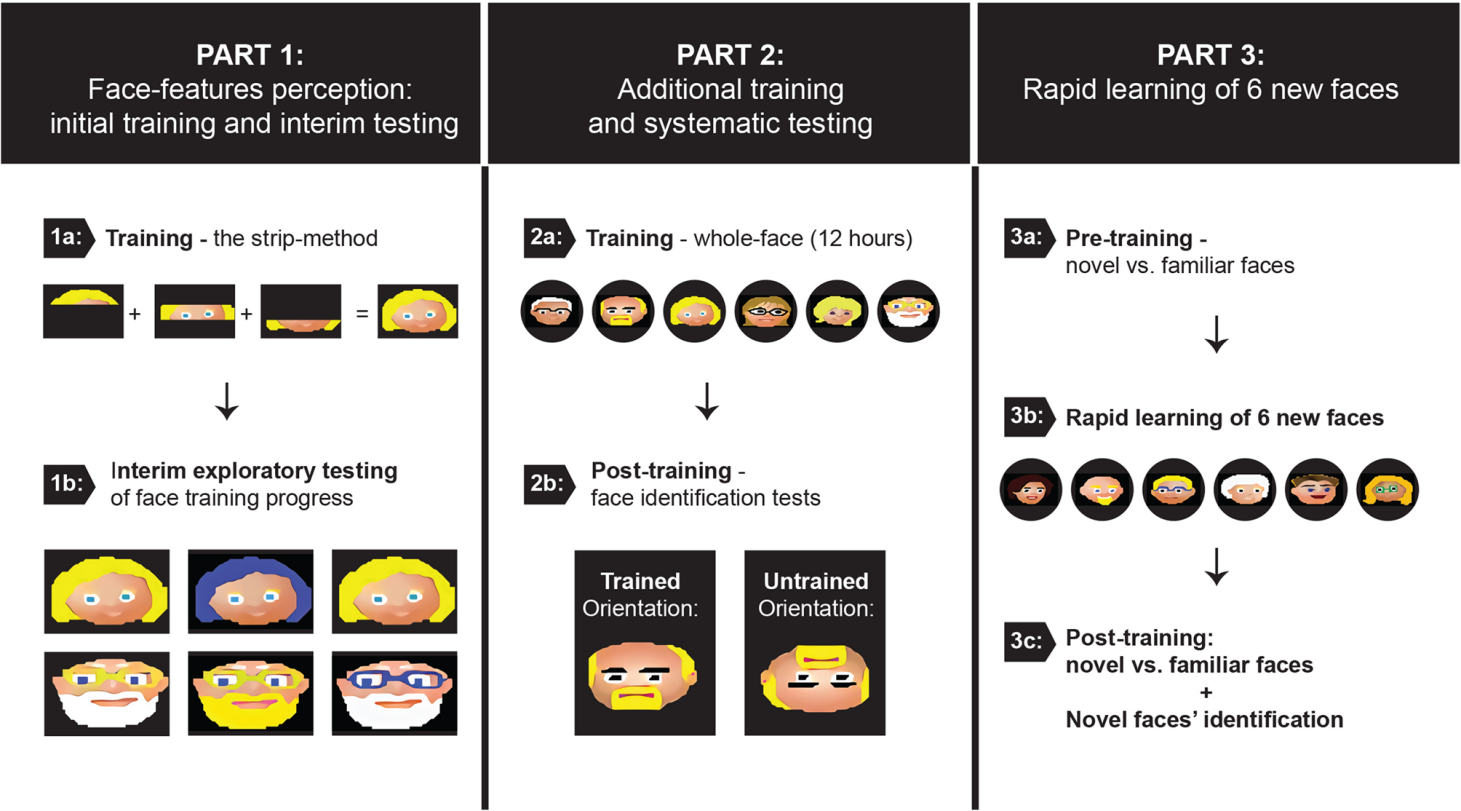
Experimental paradigm. The experiment was divided into three parts. First, participants underwent six hours of training, followed by interim testing of face identification and detection of changes to facial features (**1b**). The strip-method was used in this first part: each face was divided into three horizontal strips representing the upper, middle, and lower parts of the face (**1a**). To teach participants the composition of face parts and how to tune their hearing to perceive multiple tones at the same time, they were trained to perceived details within each strip. Following completion of this stage, participants were able to integrate their knowledge and perceive the complete face soundscapes. Second, participants underwent additional 6 h of whole-face training (**2a**). They subsequently took part in several tasks designed to test face identification, including the identification of learned faces in the untrained, inverted orientation (**2b**). Third, participants underwent an additional pre-post experiment, in which they quickly learned a new cohort of six faces and were tested on face identification before and after two hours of training (**3a**–**3c**).

**Figure 3:**
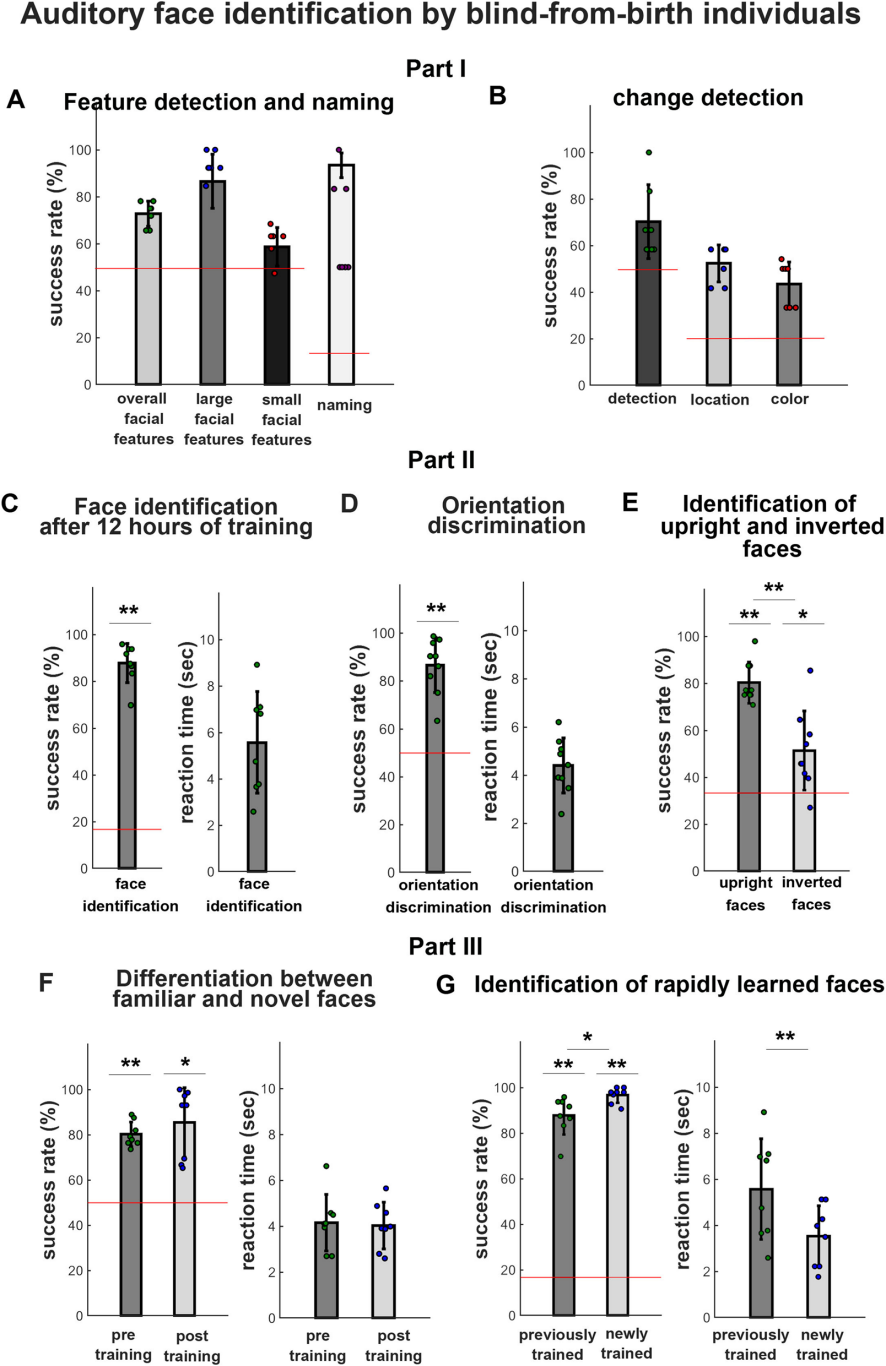
Auditory face identification by congenitally blind adults. Part I: Interim exploratory testing of face training progress (after 6 h of training): (**A**) participants were requested to answer yes/no questions on the trained faces regarding facial features (e.g., does this character have blue eyes?) to test their ability to extract meaningful information regarding facial features. Results show that participants could correctly identify facial features, especially larger features. In addition, participants were also asked to name once each trained character, and were successful in doing so. (**B**) To further assure participants’ were able to detect colorful features within the complicated soundscapes, we changed some features of the trained faces (e.g., eye-colors, hair color etc.). Congenitally blind participants were relatively accurate to detect the presence of a change, but were less accurate when asked to localize the change (i.e., to identify the modified face feature) and to identify the changed color. Part II: Face identification tests following 12 h of training: (**C**) Participants were tested on face-shape identification of the 6 trained characters. Results show that they were able to correctly identify face-shapes with high accuracy, which was significantly higher than chance level (left bar graph). In addition, they were able to provide an answer within 2 soundscape’ repetitions (right bar graph). (**D**) Participants were able to successfully discriminate upright (trained) from inverted (untrained) faces with an accuracy significantly above chance level (left bar graph). In addition, we also show that it took them less than 2 soundscape’s repetition to provide an answer (right bar graph). E. Participants were asked to identify face-shapes in their upright (trained) and inverted (untrained) orientation, which resulted in a completely different soundscape. Results show that participants were able to identify untrained inverted faces with an accuracy that was significantly higher than chance level, but significantly lower than their accuracy in identifying the faces when presented in the upright and trained orientation. Part III: Face identification of a new cohort of 6 characters after 2 h of additional training: (**F**) Participants were requested to differentiate the previously trained faces from those belonging to the new cohort, before and after a 2-h training session. Results show that participants were able to do it with an accuracy significantly above chance level, both before and after the 2-h training (left bar graph). In addition, both before and after training, they managed to provide an answer within 2 soundscape’s repetition (right bar graph). G. Following the 2-h training session, participants were successful at identifying newly-trained faces with higher accuracy than chance. They were significantly more accurate in the identification of newly compared to previously learned faces -accuracy level taken from Experiment 1 (left). In addition, they were also significantly faster in providing an answer when identifying newly compared to previously learned faces (right). In figures (**A**–**G**), error bars represent standard deviation. In figures (**C**–**G**), **p < 0.005, while *p < 0.05.

In addition to asking simple yes/no questions on the face-shapes soundscapes, we further assessed participants’ ability to extract image details by embedding changes in the features’ colors within the trained faces. For instance, changes included a change in hair color, glasses color, eye color (see Fig. 2 for some examples). We asked participants whether they could detect the change, and then to localize it to a specific facial feature within the image (hair, glasses, etc.), and finally, to identify the new color of the changed feature. Verbal responses were registered. Participants were able to identify the change in 70.24% of the soundscapes (SD =  ± 15.85%, chance level 50%), to localize it to the correct feature/location with an accuracy of 52.38% (SD =  ± 7.93%, chance level 20%), and to identify the new color with 43.45% (SD =  ± 9.58%, chance level 20%) accuracy (Fig. 3B).

### Part 2: Face identification tests following 12 h of training (n = 9)

#### Experiment 1: Face identification

In this task, participants were presented with a face-shape soundscape and asked to identify it (i.e., by telling his/her name). Results showed that following ~ 12 h of dedicated face-shapes training, participants were able to identify face-shapes with 87.38% (± 7.96%) accuracy, significantly above chance level (16.67%, *t(8)* = 26.53, *p* < 0.001, *d* = 8.76) (Fig. 3C). When analyzing reaction times, we observed participants identified a face in 5.44 s, on average (about two face presentations; SD =  ± 2.08 s) (Fig. 3C).

#### Experiment 2: Upright vs. inverted (untrained) orientation discrimination

In this task, participants were asked to discriminate between the soundscapes of upright trained faces and soundscapes of the same faces presented in the inverted (untrained) orientation. They provided their answer via response keys. Results showed participants could correctly classify the presented face-shape’s orientation as either upright or inverted, in 86.56% (SD ± 11.54%) of the trials. A t-test against chance level (50%) confirmed they could classify a face’s orientation significantly above chance level (*t(8)* = 9.50, *p* < 0.001, *d* = 3.05). When analyzing reaction times, we observed participants classified face orientation in 4.40 s, on average (about two face presentations; SD =  ± 1.14 s) (Fig. 3D).

#### Experiment 3: Identification of upright and inverted faces

In this task participants were requested to identify face-shapes presented in both upright and in the inverted orientation, and provide their answer via response keys. Our results confirmed that participants were able to identify faces in the upright orientation, with an average accuracy of 80.32% (standard deviation (SD) ± 8.7%), significantly above chance level (30%, *t(8)* = 16.28, *p* < 0.001, *d* = 5.44). They were also able to identify characters when presented in the untrained, inverted orientation, with an average accuracy of 51.39% (SD ± 16.86%), again significantly above chance level (30%, *t(8)* = 3.27, *p* = 0.006, *d* = 1.09). Finally, participants were significantly more accurate at identifying upright than inverted characters (*t*(16) = 4.78, *p* < 0.001, *d* = 1.59) (Fig. 3E).

### Part 3: Face identification of six additional characters after 2 additional hours of training (n = 9)

#### Experiment 4: Discrimination of first cohort vs. second cohort of trained faces before and after 2 h of additional face training

Before and after the additional 2-h training, participants were presented with both cohorts of faces and were asked to discriminate the soundscapes as either belonging to the first (previously trained) or second cohort (newly trained) of learned faces. Participants provided their answer via response keys. Before training, participants successfully discriminated trained from novel faces with 80.2% (SD ± 5.5%) accuracy. A t-test against chance (50%) confirmed performance was above chance level (*t*(8) = 15.55, *p* < 0.001, *d* = 5.50), indicating the auditory properties of the novel faces were distinctive enough to enable their discrimination from the previously trained cohort of faces (Fig. 3F).

When we repeated the same experiment after training, we observed a trend of improved discrimination between the two cohorts of faces; success rate was 85.4% (SD ± 15.4%), significantly above chance level as well (50%, *w* = 36, *p* = 0.014). However, comparing performance before and after training revealed no significant training effect (*t(8)* = 1.14, *p* < 0.29, *d* = 0.40). Namely, discrimination performance did not significantly improve following training. Similarly, participants were not significantly faster in discriminating previously learned faces from the cohort of newly-trained characters presented during the 2 h of additional training. Discrimination took, on average, 4.16 s (SD ± 1.23 s) before training and 4.04 s (SD ± 1.02 s) after training (*t*(14) = 0.56, *p* = 0.30, *d* = 0.20) (Fig. 3F).

#### Experiment 5: Identification of six additional characters trained for 2 h

In this task, participants were presented with the set of newly trained face-shapes and asked to identify all exemplars via verbal responses. Results showed participants could successfully identify all recently learned faces, correctly identifying 96.74% (SD ± 3.36%) of the faces, on average, significantly above chance level (16.67%) (*t*(7) = 74.45, *p* < 0.001, *d* = 25.71). Participants were not only accurate; they were also quick in identification, and did so in 3.52 s, on average (SD ± 1.33 s) (Fig. 3G).

Finally, to investigate learning dynamics, we compared the accuracy rate of the identification of previously learned faces (from Experiment 1) with that of novel faces (from the current experiment) using a paired-samples Wilcoxon signed-rank test (see “Methods”). This test revealed a significant effect (*w* = 36, *p* = 0.014)), whereby participants were more accurate in identifying novel faces learned only for 2 h, than they were for the first set of learned faces (i.e., learned within 12 h of training). In addition to being accurate, participants were also significantly faster in identifying these newly trained faces compared to the previously learned set of faces (*t*(14) = 4.06, *p* = 0.005, *d* = 1.43) (Fig. 3G).

## Discussion

In this work, we show that congenitally blind adults could successfully identify colorful face-shapes exemplars via the auditory modality, despite life-long minimal experience with such shapes, following a tailored and engaging ~ 12-h training program with a visual-to-auditory Sensory Substitution Device (SSD). Moreover, we show that blind individuals were not only able to identify SSD-presented trained face-shapes, but quite strikingly, could also generalize face-shape identification to the same faces presented in their untrained, inverted orientation. The identification of face images in their inverted orientation requires the perception of a sound with completely different spectral properties than those of the trained, upright corresponding exemplar, potentially paired with the mental rotation of the face-shape representation. Finally, we show that the comprehensive 12-h training program with the original cohort of six faces enabled participants to rapidly learn (i.e., after 2 additional hours of dedicated training) a new set of six face-shapes. The colorful images of faces used in our study represent the most complicated category of images systematically trained using SSD to this point. This work has implications in the fields of visual and auditory processing, learning dynamics, mental imagery, and rotation, as well as practical implications for rehabilitation of blind and visually impaired/restored individuals, as discussed below.

### Face-shape identification via the auditory modality

In this work we show that despite the lack of life-long extensive experience with face-shapes, congenitally blind adults can learn to perceive such shapes via the auditory modality and coherently organize this auditory perception to allow the identification of face-shapes exemplars.

To enable the learning of face images via auditory SSD, we developed a tailored training program that aimed at maximizing the learning of visual spatial face-feature configuration. Specifically, we avoided teaching facial features separately, as can be done through touch. Rather, we presented our face stimuli using the strip-method; for each face-shape, we created three horizontal strips, one for each of the uppermost, middle, and lowermost sections of the face (Fig. 2). Then, to promote participants’ engagement with complete face exemplars, during training, participants were encouraged to discuss facial features and their spatial arrangement within the faces. Arguably, the use of this spatial configuration approach to face-shape learning may have allowed participants to better integrate the face parts and build a “mental image” of the complete face object, and this, in turn, may have facilitated recognition of individual exemplars^20^. However, to assure that a manipulable “mental image” of the soundscape-face is really constructed, future studies should specifically investigate whether properties of face-shape perception known in the sighted population, extend to the blind population perceiving face-shapes via SSD (e.g., changing in view-points), and whether to perceive auditory face-shapes participants rely more on object-based mechanisms or utilize approaches more typical of visual face perception, such as holistic face processing^21^. Recently it has been shown that in some instances, interaction with human voices or tactile face-shapes recognition activate regions within the visual neural face recognition system^22,23^. Future studies should explore whether the case of face-shape recognition via the novel and non-ecological stimuli provided by the auditory soundscapes, tackles on object related, or rather, face related modules in the deprived visual cortex.

Notably, studies on visually restored individuals show that even after the restoration of functional vision, neural tuning to faces remains impaired^2^. For instance, although following cataract removal surgery, children can discriminate face from non-face exemplars^24^, a study on congenital cataract reversal surgery in children, showed the absence of the distinguished N170 marker for visual faces^25^. Functional Magnetic Resonance Imaging (fMRI) studies corroborate electrophysiological findings by showing individuals’ lack of functional selectivity to faces following prolonged visual deprivation^26–29^. Together, these results suggest that visual restoration may not be sufficient to establish accurate face-shapes recognition in sight-restored individuals. However, our results may encourage the hypothesis that SSD targeted rehabilitation, using dedicated and specific training, might be introduced after sight restoration to aid visual perception ultimately maximizing restoration outcomes^30,31^.

Moreover, following the completion of the training program, our participants were significantly quicker when learning to identify new exemplars belonging to the same group of objects (i.e., face-shapes), indicating that the perceptual concepts learned during training could be quickly exploited to learn novel stimuli. This result may further encourage the introduction of SSD-based training as a rehabilitation tool for the blind as well as aid in sight restoration rehabilitative programs, by suggesting that with increased exposure, SSD-related learning time can become shorter. Future studies should further investigate the mechanisms mediating this enhanced performance, including the generalization of trained principles to novel stimuli as well as the role of memory during the learning process. Indeed, we cannot exclude that our blind participants may have learned the main distinct features of the shortly-trained face-shapes soundscapes, and may have used them to memorize the soundscapes.

### Perception of multi-color, complicated images via the auditory modality

The translation of colorful cartoon faces via the EyeMusic SSD into the auditory modality results in complicated soundscapes. Because of the sweep-line technique used by EyeMusic and other visual-to-auditory SSDs^12^, up to 30 musical tones of different timbres representing up to 30 visual pixels are played in sync. However, our results suggest that a training program aimed to specifically teach the perception of multiple pixels at the same time, namely one of the biggest challenges of visual-to-auditory SSD perception^19,32^, can result in good integrational ability. This was demonstrated by participants’ successful recognition of complicated face-shapes soundscapes, as well as by their detection of details embedded within the soundscapes that we assessed during training. This result suggests that to improve the usability of SSDs for the blind, training programs with specific objectives and dedicated training approaches should be designed to achieve specific rehabilitation outcomes.

One additional important aspect of these results is that they suggest that color, in the form of color-to-timbre mapping, may have exerted a facilitatory role on the learning of these complex soundscapes. Indeed in vision, color has been found to enhance functional acuity or in other words, helped in solving complex visual tasks^33^. Indeed in the context of visual tasks, the identification of low-resolution objects is enhanced by the addition of color and specifically observed to facilitate face recognition^15^. Even though these visual results cannot be directly projected to the current (auditory) condition, we propose that our auditory results suggest that color transformation aided our participants in the correct perception of all face parts, and ultimately of whole face-shapes. Specifically, we suggest that the addition of the ‘color’ feature to soundscapes might have provided another discrimination feature among auditory pixels, and thus might have increased discriminability among face features. Crucially, these results are in line with another recent work from our team which similarly showed that color-to-timbre mapping enhanced discrimination of auditory letters and boosts reading performance via the EyeMusic SSD compared to identical monochrome soundscapes^16^.

Future studies exploring the advantages of auditory SSD-conveyed colors by directly comparing colorful to monochromatic complicated soundscapes will advance our understanding of the extent to which the visual domain compares to the auditory domain in image perception.

### Face-shapes identification in the inverted (untrained) orientation

The successful identification of face-shapes in their untrained, inverted orientation suggests participants were able to build a mental representation of the visual information embedded within a soundscape that they could manipulate (e.g., rotate) to match its upright and trained orientation. The process by which participants were able to generalize face identification to the inverted orientation could arise from two non-mutually-exclusive mechanisms: first, the extraction of features within the inverted face (blue eyes, long, blond hair etc.) and the matching of the features to the suitable upright face via feature-based matching; second, the mental rotation of the whole face-shape with its different spectral properties and the consequent matching to the corresponding face in the upright orientation. Mental rotation of objects is a skill that develops through life, with indications of its facilitation via visuo-haptic object manipulation^34,35^. Mental rotation of objects has been linked to motor experience, hormones, and experience-dependent processes^36–38^. Previous studies showed that blind individuals, even those who acquired blindness at birth and thus have no visual experience, are able to mentally rotate objects perceived through touch and are not deficient in their mental rotation abilities compared to sighted adults performing the same task also through touch^39–41^. Overall, the current results strengthen previous findings concluding that both the construction of a mental image and its rotation are not limited to information acquired via the visual modality. Yet unlike the tactile modality which is commonly used for tactile recognition of objects by both sighted and blind, the auditory sensory modality does not commonly provide information about objects’ shapes or orientation. Our study thus expands previous findings by providing initial results suggesting that the building of a mental image and its complex spatial manipulation can also be achieved via the non-traditional auditory modality.

Interestingly, studies on face identification in sighted individuals show, similarly to the present results, that the identification of a face in its inverted form results in a drop in both accuracy and identification speed termed the “face inverted effect”^42^. Such an effect has been shown to be especially enhanced when configural aspects of the faces were manipulated (i.e., when faces differed among themselves in terms of spatial features such as the spacing between nose and mouth) compared to featural or contour manipulations^6^. Future studies should investigate whether SSD face identification in the blind is based on the same mechanism by also employing traditional paradigms typically used in the sighted population to investigate face inversion, such as the composite effect^21^. These works will advance our understanding of the processes underlying SSD face-shapes learning and generalization. In addition, and more generally, such studies will advance our understanding of the properties and constraints characterizing orientation-manipulations via the auditory modality.

## Conclusions

In this work, we showed for the first time that congenitally blind adults can successfully perform whole-face-shapes identification via the auditory modality using a shape and color preserving visual-to-auditory SSD. We show that in the context of a dedicated training program, congenitally blind adults are able to perceive complicated soundscapes, and despite the auditory complexity of the sound, they are able to perceive detailed features embedded within the image and use this information to correctly identify face-shapes. Moreover, although shape identification and its orientation in space are not commonly performed using the auditory modality in daily life, and despite the completely different acoustic properties of the untrained, inverted face-shapes compared to the trained, upright faces, we found that congenitally blind adults are able to generalize face-shape identity to the inverted (untrained) orientation. Finally, we show that once the initial intense 12-h face training was concluded, our SSD-expert participants were able to extend the concepts learned during training to a new cohort of faces; they could successfully learn a set of new face-shapes within 2 h of additional tailored training.

On the behavioral front, our results show the feasibility of the use of SSD to convey complicated visual objects. On the clinical front, our results encourage the use of SSD as a rehabilitative tool both for the blind and for the visually restored populations. On the neural front, our results produce interesting hypotheses for face processing, auditory processing, and cross-modal plasticity in the congenital absence of visual input.

## Methods

### Participants

12 congenitally blind participants (four men, average age 36 years) with no reported neurological conditions participated in the experiments. All participants had extensive experience (50–70 h of training) with EyeMusic or other visual-to-auditory SSDs before starting the experiment. For detailed characteristics of the participants, see Table 1.

**Table 1:** Demographic details of participants.

Participant	Age	Blindness cause	Light perception	Age at blindness onset	Braille reading	Handedness	Participation in Part I: Interim testing	Part II: face identification	Part III: rapid face training
FO	33	Microphthalmia	No	0	Yes (since age 5)	Right		V	V
ElMa	36	Retinopathy of prematurity	No	0	Yes (since age 5)	Right	V		
JH	42	Leber’s disease	Faint	0	Yes (since age 5)	Ambidextrous	V	V	V
NN	45	Retinopathy of prematurity	No	0	Yes (since age 6)	Right	V	V	V
PC	41	Retinopathy of prematurity	No	0	Yes (since age 6)	Right	V	V	V
PH	42	Rubella	No	0	Yes (since age 5)	Right		V	V
FN	33	Leber’s Disease	Faint	0	Yes (since age 5)	Right		V	V
DS	34	Retinopathy of prematurity	No	0	Yes (since age 6)	Right		V	V
UM	39	Retinoblastoma	No	3	Yes (since age 4)	Ambidextrous	V		V
HB	27	Anophtalmia, fall	No	< 1	Yes (since age 4)	Left	V	V	V
UM	33	Anophtalmia	No	0	Yes	–	V		
DK	27	unknown	No	No	Yes (since age 4)	Ambidextrous		V	

The Hadassah Medical Centre Ethics Committee approved the experimental procedure; written informed consent was obtained from each participant. All methods were performed in accordance with the relevant guidelines and regulations. Participants were reimbursed for their participation in the study. The participant who was involved in the movie demonstrating auditory face identification provided their informed consent for the publication of identifying information on an online open-access platform. Notably, and in accordance with the informed consent process, participants could decide to participate in only a subset of the experimental procedure without providing us with a reason. Please note that since not all participants took part in all aspects of the training program, we report the number of participants that performed each part of the experimental protocol.

### Tools

#### EyeMusic algorithm

The EyeMusic visual-to-auditory SSD was used to teach participants to identify whole-face shapes, as well as words and hand gestures (see details in the following section). EyeMusic transforms each pixel of a given image into what we term auditory soundscapes, an auditory pattern preserving shape, color, and spatial layout of objects. In brief, the EyeMusic algorithm down-samples each image to 50*30 pixels. Then, a sweep-line approach transforms each pixel in a given image into a corresponding sound. First, the x-axis is mapped to time; each image is scanned column-by-column from left to right, such that pixels on the left side of the image are played before those on the right. Second, the y-axis is mapped to pitch variations using the pentatonic scale, such that the lower the pixel in the image, the lower the corresponding pitch sonifying it. Third, color is conveyed through timbre manipulations, such that each color is played using a different musical instrument. EyeMusic has five colors (white, green, red, blue, and yellow), and black is mapped as silence (Fig. 1). Brightness levels are conveyed via sound volume variations (for full details, see Abboud et al.^11^). During both the training and experiments, Eye-Music soundscapes lasted 2.5 s.

#### EyeMusic training sessions

Face identification via shape recognition is a visually-dominant skill with which congenitally blind adults have little if any experience. Before training, our congenitally blind participants were largely unaware of face-related shapes; thus, a crucial aspect of our structured training program was familiarization with this novel object category. Furthermore, auditory face identification is an extremely challenging behavioral task because of the complexity of the soundscapes created by faces. Therefore, our training program focused on how to interpret such complex auditory soundscapes, specifically the perception of multiple tones played at sync.

Participants in all experiments were already proficient EyeMusic users who had extensive previous training with the device (> 50 h) before face training began. Importantly, unlike previous training procedures which consisted mostly of line drawings of simple shapes, this was the first time participants were presented with images filled with color and with colorful features embedded within the images whose interpretation was crucial to succeed in the tasks. The difference between line drawing and color-filled images, when translated to EyeMusic, is a difference between the perception of 1–2 tones and the perception of 20–30 tones, with differences in timbres between tones reflecting colorful facial features. Through the training program, participants learned to extract the meaningful differences among tones played at the same time and to construct a comprehensive perception of the image through its soundscape.

### Experimental procedure

#### Part 1: Face-feature perception—initial training and interim (explorative) test

EyeMusic experts underwent six hours of face training focused on the learning of common facial features and the perception of complicated full-face soundscapes. At the end of this first training, a subset of the cohort (seven participants) took part in an interim face-feature identification test to check and quantify their facial-feature perception to ensure the efficacy of the training program and face-perception progress.

#### Part 2: Additional training and systematic testing

The second part of training, consisting of six additional hours, focused on the identification of six cartoon faces. This part of the training focused on participants’ ability to identify each whole face and notice details embedded within the face when it was played as a whole. At the end of training, a subset of the cohort (nine participants, five of whom also took part in the interim test at the end of Part 1) took part in four experiments testing face identification, face-feature detection, discrimination of faces between the upright (trained) or inverted orientation (untrained), and identification of faces in the inverted orientation (untrained).

#### Part 3: Rapid learning of six new faces

To test whether principles learned during the face-training program could be easily generalized to the study of additional face exemplars, a subset of nine participants from the cohort participated in an additional experiment in which they learned six new cartoon faces during 2 h of additional training. Testing was repeated before and after training on the discrimination of trained vs novel, untrained faces and on the identification of newly trained faces (Fig. 2).

### Training procedure and experiment

#### Part 1: Face-feature perception—initial training and interim (explorative) test

##### Part 1a: Face training

To address the soundscapes’ complexity, the first part of the training program consisted of three sessions of two hours each, specifically designed to teach participants the common details of faces and the perception of complex soundscapes. First, participants were introduced to six cartoon faces adapted from the children’s game “Guess Who” and translated into soundscapes using EyeMusic (see Fig. 1). In the first stages of the training, and for each face separately, participants learned to interpret separate horizontal strips of the images (bottom, top, and middle), to gradually advance their skill so they could focus on perceiving small details embedded within the complex sounds.

In more detail, each visual image of a cartoon face was cropped into three strips—top, middle, and bottom portion of the image (Fig. 2). Participants were instructed to listen carefully to the first strip, the top strip, and to detail as much as possible the colors and shapes embedded within the strip. Once details were given, the participant and instructor discussed the details’ possible implication for the face, such as: “Could the color you heard represent hair? Or is it skin-color that represents a bald head? Can hair be heard all along the strip, or only at the beginning and the end, representing a bald patch?” Then, the middle portion of the face was played; once again, the participant was requested to detail as much as possible the different tones within the strip. With the help of the instructor, the participant was instructed to concentrate on specific parts of the image, such as notes representing eyes, and in some cases, the absence of notes. The bottom strip was played in the same way. After participants learned all three strips, the complete soundscape was introduced, and participants were instructed to integrate the details from the strips into the perception of the complete soundscape. With the help of the instructor, and by comparing the complete soundscape to the strips, participants were able to “hear” the features they learned to identify in the strips, while listening to the complete soundscape. During the presentation of face-shapes we introduced and discussed in more depths some concepts related to face-shapes participants had only brief and descriptive knowledge of. For instance, the concept of eyebrows and their location as framing the eyes, the components of the eyes such as the pupils, the fact that irises are a round shape within the larger eye and differ in color among different people (and on top of that, that the colorful part of the eye, contains another round, black shape—the pupil), that eyebrows and hair usually share color, the general difference in eyebrows thickness between men and women, the fact lips are a distinct facial feature, thanks to their contrasting color from the skin of the face, the relation between glasses and the eyes (the transparent lenses with a colorful frame). These concepts were in general unfamiliar or did not play a role in participants’ lives before the experiment. These discussions raised some interesting questions from our participants, and also connected them to some social norms and conventions (such as the perception that a light eye-color is considered unique and beautiful in Israel; the idea of women trimming their eyebrows; the use of makeup to elongate eyelashes and make lips “pop” against the skin; coloring ones’ hair etc.).

In this part of training, participants, with the help of the instructor, also concentrated on the spatial relationship between face parts. Although they had the semantic knowledge on the position of the eyes “above” the nose, and the mouth “below” the nose, the use of three horizontal strips, rather than separate isolated features, facilitated and strengthened the understanding of this spatial configuration. Indeed, we qualitatively observed that with each additional learned face, participants grew more comfortable in their skills and could more quickly describe the exemplar.

After participants were comfortable with their ability to perceive features and could describe in detail the face they were hearing, the instructor provided a name for the just-learned face. This was done to limit the possibility that the participant would simply match an abstract sound to a name rather than perceive the soundscape with its details. Notably, although during this phase we presented portions of faces, we never trained single facial features in isolation: each strip contained multiple facial features or parts of features (e.g., each top strip contained hair, eyes, glasses etc.). This is very different from tactile exploration where each facial feature is generally explored alone, separately from the others. We assigned a name to the cartoon because it has been shown that assigning names to new faces improves face recognition skills^43^. In addition, doing so gave participants with a sense of achievement; they could recognize specific faces that had been inaccessible before training. After three 2-h sessions, participants completed the interim test.

##### Part 1b: Interim explorative test after six hours of face training

**Assessment 1: “Guess Who” game.** We performed an interim test to qualitatively monitor face-training progress. Using the “Guess Who” children’s game, we asked each participant yes/no questions on the facial features of each of the six faces, for example: “Does this character have blue eyes?” “Does this character wear glasses?” The purpose of this test was threefold. First, we wanted to confirm the training was effective, and participants had learned to identify facial features embedded in the soundscapes. Second, we wanted to ensure the participants had learned and focused on all facial features, thus preventing face recognition based on a simple sound-to-name matching, Third, we wanted to give participants an opportunity to apply their acquired skills to a more ecological and fun activity they were unable to participate in prior to training—the blind are generally excluded from this popular children’s game.

This experiment included 32 trials, containing yes/no questions about the faces, plus six naming trials (one naming trial per cartoon face). The questions addressed both large visual facial features (16 questions about hair, etc.) and smaller facial features (16 questions about eyes, glasses). Stimuli were presented using the EyeMusic SSD, and each stimulus was played until an oral response was given by the participant. Responses were registered by the experimenter. Rate of correct responses for yes/no answers was analyzed. As a secondary result, the percentage of correctly identified characters was also assessed.

**Assessment 2: Change detection**. To further test participants’ ability to recognize features embedded within the complicated soundscape, we performed a “change detection” task in which the color of one feature of the image was changed (e.g., the eye-color; hair color, etc.). Our aim was to see how well facial features embedded within the soundscapes were detected by participants by testing whether they were able to detect changes made to these features.

This experiment included only 12 trials. We limited the number of trials because only a limited number of facial features and colors could be manipulated in a meaningful way. We only included color-related manipulations. Participants were allowed to listen to the original soundscape and the manipulated soundscape to compare them before giving their answer. This task was very intense and challenging for participants, as it required a lot of focus to answer correctly, given the complexity of the soundscapes and the small manipulations we introduced.

Stimuli were presented using the EyeMusic SSD. Each stimulus was played, either continuously or in an interwoven fashion with the original soundscape, until participants provided a response. Specifically, participants were asked to respond on whether they detect a change between the two soundscapes. And if yes, to localize the change (i.e., in which face feature) and identify the changed color. Responses were registered by the experimenter. We assessed the accuracy of detected changes, the accuracy of localizing the changes (how accurately participants identified the specific feature that was changed, or the location of the change within the spatial soundscape), and the accuracy of identifying new colors.

#### Part 2: Additional training and systematic testing

##### Part 2a: Face training

By the end of Part 1 of training, participants were largely able to answer yes/no questions on the faces and identify changes in color of the features within the learned faces. Yet because of the complexity of the soundscapes, participants were not proficient at identifying the faces or discriminating among faces with similar visual features (e.g., long-haired women). Thus, the second part of training, consisting of three additional two-hour sessions, focused on the perception of complete soundscapes of faces using several training tasks. One task was to identify each complete face, without first listening to the strips composing it. Another was to listen to two faces played in an interwoven manner and identify both faces. This task helped participants note specific differences between similar faces. For instance, the three presented women had similar hair color; thus, to distinguish among them, participants had to focus on the sound amplitude (volume) representing how bright or dark each woman’s hair was. In this part of training, participants were encouraged to openly describe the faces.

##### Part 2b: Testing face identification and generalization to untrained orientation (inverted) after 12 h of training

All experiments in parts 2 and 3 were performed using Presentation software (Version 18.0, Neurobehavioral Systems, Inc., Berkeley, CA, www.neurobs.com).

**Experiment 1: Face identification**. First, to ensure success of training, especially participants’ ability to identify each of the learned six faces, we conducted a basic face identification experiment. Participants were asked to name each of the six experimental faces they received training on. Each character was played repeatedly until participants named it (roughly within two repetitions). Each character was presented in 16 separate trials, 96 trials overall, presented in random order. Participants provided their responses verbally, and the experimenter inserted them in the computer. The rate of correct responses and reaction times were analyzed.

**Experiment 2: Upright vs. inverted (untrained) orientation discrimination**. Second, to ensure training had taught participants to understand face shapes and composition and to test whether participants were able to recognize face orientations that were not trained (i.e., inverted), we conducted an orientation-discrimination task. During the discrimination task, a character was repeatedly played in either the standard upright (trained) or inverted (up-side down) orientation (untrained) until participants assigned an orientation to the soundscape. Participants used two response keys to register their answers (either upright or inverted). Each character in each orientation was presented six times (six separate trials) for a total of 72 trials presented in random order. The rate of correct responses and reaction times were analyzed.

**Experiment 3: Identification of faces in upright and inverted orientations**. This experiment was conducted while participants were lying in an MRI machine. We wanted participants to be naive to the identification task of inverted faces and perform it for the first time while being scanned, Therefore, we conducted this experiment in the scanner. Note that our neuroimaging results will be reported in a separate paper. In vision, the inversion of a face does not change the visual attributes of the face itself, only their position in space, but in the auditory modality, the inversion of a face completely changes the acoustic properties of the soundscape. The ability to identify characters in their inverted orientation would show that participants were able to recognize characters based on their unique characteristics, not by simply matching sound to an arbitrary name, and could also perform mental rotation of the soundscapes’ representations.

We used four sets of face stimuli: *trained faces, trained faces in the untrained, inverted orientation (inverted faces), entirely new, untrained faces (new faces),* and *scrambled faces. Trained faces* included the six colorful faces participants learned to identify during training. *Inverted faces* included each of the six trained faces sonified using EyeMusic in the untrained, inverted (upside down) orientation*. New faces* included six visually similar faces not introduced during training. *Scrambled faces* included the six familiar faces in a scrambled form. The faces were divided into nine parts and scrambled randomly using MATLAB. The resulting images were sonified via EyeMusic. The scrambled faces condition was included as a motor control, and we do not discuss results for this condition in this paper.

The conditions were presented in separate blocks. Each condition was repeated six times, in a pseudorandom order, for a total of 24 blocks. The experiment was repeated for four runs, for a total of 96 blocks. Each exemplar was repeated eight times. In each block, two different stimuli belonging to the same experimental condition were displayed, each lasting five seconds (two consecutive repetitions of 2.5 s per stimulus), followed by a response interval of 2 s. Each block started with an auditory cue indicating the tested category and lasting two seconds (trained faces, inverted, new, scrambled). Participants provided their responses using a response box at the end of both repetitions of a stimulus. For trained (upright) and inverted conditions, participants were instructed to identify the character. To limit the number of response keys thus allow one-handed response, in two of the four runs, only female characters were presented, while in the other two runs, only male characters were presented. As a result, chance level in this section is 30%. In total, each block lasted 16 s and was followed by a 10 s rest interval. For new faces, participants performed a feature detection task; they were instructed to listen to each soundscape carefully. If they could identify yellow features, they were instructed to press one button, and if not, to press another button. Digital auditory soundscapes were generated on a PC, played on a stereo system, and transferred binaurally to participants through a pneumatic device and silicone tubes into commercially available noise-shielding headphones. For all experiments, accuracy of responses was analyzed. In this work, we only present results for the trained and inverted conditions. The rest of the results from the scanning session will be presented elsewhere.

#### Part 3: Rapid learning of six unfamiliar faces

##### Part 3a: Learning of new faces

Following the in-depth learning of the six faces, we wanted to test whether learning of new face examples would become faster. We thus taught participants to recognize six unfamiliar faces, in two hours of training.

##### Part 3b: Assessment of learning

To assess learning, we performed the following experiments.

**Experiment 4 (pre-post training)**. This experiment involved the discrimination of previously trained faces vs. unfamiliar faces (untrained). Before training, we tested participants on a discrimination task of all 12 faces; six were already familiar from previous training, and six were new. Participants were asked to classify if a face belonged to the cohort of familiar faces (i.e., trained faces) or not (i.e., untrained), and provide their response by pressing one of two possible response keys with the index and ring fingers. Each face was repeated continuously until a response was provided. Each face was repeated six times for a total of 72 trials presented in random order. The number of correctly classified faces and reaction times were analyzed. This task was performed to ensure that despite deliberate visual similarities among the familiar and unfamiliar faces, the resulting soundscapes were different enough to be distinguished by our participants.

We repeated the experiment to assess improvement in discrimination following training on the six new faces. The experiment was the same, but this time, participants were instructed to discriminate each face as belonging to either the “old cohort” or the “new cohort” learned that day.

**Experiment 5**. We conducted a face identification task with the six newly-trained faces. Participants named each of the six characters learned during the final 2-h face-training session. Each character was played repeatedly until participants named it (roughly within two repetitions). Each character was presented in 12 separate trials, 96 trials overall, in random order. Each face soundscape was played continuously until a response was provided. Participants were instructed to press the space bar when they knew the answer and then to provide their responses verbally. The experimenter entered responses into the computer. The rate of correct responses and reaction times were analyzed.

### Data analysis

Data were analyzed using JASP (version number 0.14). We used a paired t-test when comparing results for different conditions (upright/inverted or pre-post,) and a t-test against chance level when comparing conditions to chance. Chance level was set at random, since participants had no previous experience with face soundscapes, and were unable to interpret the soundscapes, or even extract meaningful information regarding the larger features prior to training. We also provide an independent assessment of face identification via face-parts using an SVM model (see “Supplementary material”). Data was checked for normality using the Shapiro–Wilk test. In all but two instances, the data was normally distributed. In the two instances in which the data deviated slightly from normality, we used the non-parametric Wilcoxon Signed Rank test. The first instance is comparing identification rate of familiar vs novel faces. The second instance, is the post training discrimination of familiar vs novel faces.

Part 1 was an interim, explorative test; we provide only a descriptive report of the results, given the low number of data points acquired.

## Supplementary Information

Supplementary Information 1.

Supplementary Video 1.

Supplementary Information 2.
