# Supplementary material for: Congenitally blind adults can learn to identify face-shapes via auditory sensory substitution and successfully generalize some of the learned features
Source: Sci Rep. 2022 Mar 14;12:4330. doi: 10.1038/s41598-022-08187-z (PMC8921184; doi:10.1038/s41598-022-08187-z)
Supplement: Supplementary file 3 — Supplementary Information 2. [file 41598_2022_8187_MOESM3_ESM.docx]

SVM estimation of soundscapes discriminability

Due to the complexity of the face soundscapes, we aimed at testing if there were enough discriminable perceptual characteristics of each auditory face that would allow its identification. For this aim we used three different SVM models trained on different cochleograms produced from the faces’ soundscapes and tested the ability of each model to generalize to the full faces’ soundscapes.

First, we wanted to test whether it was perceptually possible to use only parts of the whole-faces’ soundscapes to identify each character, i.e., potentially without going through specific training on the soundscapes. This was done also to exclude the possibility that our blind participaants did not learn to perceive the complex whole-face soundscapes, and instead relied on unique perceptual characteristics of each auditory face. To this aim, we created an image dataset containing all possible unique 5X5 pixels patches for each of the faces (see Fig S1, part 1). To assure we would only select unique patches, we first extracted all possible 5X5 patches from all faces, then we compared the patches produced from each exemplar among each other, and omitted any identical patch (i.e., two identical patches, taken from two different face exemplars). This results in a library of unique patches for each of the faces. These patches were labeled with the name of the face from which they were produced, given they were unique identifiers of each exemplar (each patch can only be found in one of the face-exemplars).

Then we created soundscapes of all these patches based on the Eye Music’s sonification algorithm, i.e., represented by an 8-bit resolution and a sampling frequency of 44,100 Hz. We then transformed each resulting soundscape into a cochleogram’s time-frequency representation. The cochleagram representation is modelled on the frequency-selective property of the human cochlea: it offers narrow frequency components in the lower frequency range and wide frequency components in the upper frequency range. Note that the finer frequency resolution in the lower frequency range helps to reveal more spectral information without significantly losing spectral information in the upper frequency range ^1^. The use of cochleagram has shown to be effective in audio classification and separation tasks ^2,3^. The cochleogram parameters were based on standard parameters: we set the number of gammatone filters to 30, a resample factor of 4 using a polyphase filtering with a default Kaiser window with a size of 5.

After having created a dataset suitable for training an SVM, we trained a linear SVM with standard parameters on all the cochleograms. The SVM was run via SK-learn, a machine learning library in Python. The resulting classifier, despite being trained on all unique cochleogram’s time-frequency representations of the related face-exemplars’ patches, correctly classified only one of the six face-exemplars, the equivalent of random chance level. This method suggests that generalization of face-parts to the entire soundscape is not perceptually possible. This in turn suggests that this most probably was not the approach used by our participants to learn to discriminate among different faces’ soundscapes.

We then tested the generalization ability to whole-face soundscapes’ discrimination of a second SVM model, this time trained on the cochleograms generated from horizontal “strips” similarly to the ones used in the first part of our 12-hours training. Specifically, we translated each strip into a soundscape and then transformed it into a cochleogram using the same approach detailed above (see Fig. S1, part 2). The cochleograms were labeled with the name of the face from which they were produced. After training on the “strips”, the classifier was able to generalize training to the whole-face cochleograms. These results confirm that within the “strips”, there were indeed enough discriminable perceptual information that would allow to correctly perceive the corresponding whole-face soundscape. These results in turn, confirm the validity of the “strips method” to teach complex soundscapes.

Finally, with our third SVM model we wanted to test whether the faces them-selves contained enough unique perceptual information that would allow to learn them and therefore differentiate among them. To this aim, we created a dataset of the original faces used in the experiment, but with color-changed features (e.g., different eye or hair colors). This dataset mimics the “change detection” task we describe in the manuscript, yet resulted from an automatic generation of color-changed faces (see Fig. S1, part 3). Each of these color-altered images were labeled with the name of the face from which they originated. We then proceeded with the creation of the cochleograms of these color-altered faces and applied to this dataset the same SVM model described above. The SVM was able to generalize the trained inputs to the original faces, therefore correctly differentiating among the faces. These results suggest that there was enough auditory discriminability among the whole-face soundscapes.

Note that these results only inform on whether the cochleograms used to train the SVM model(s) contained enough perceptual information that would allow to perceptually discriminate among the whole-faces soundscapes.

Figure S1


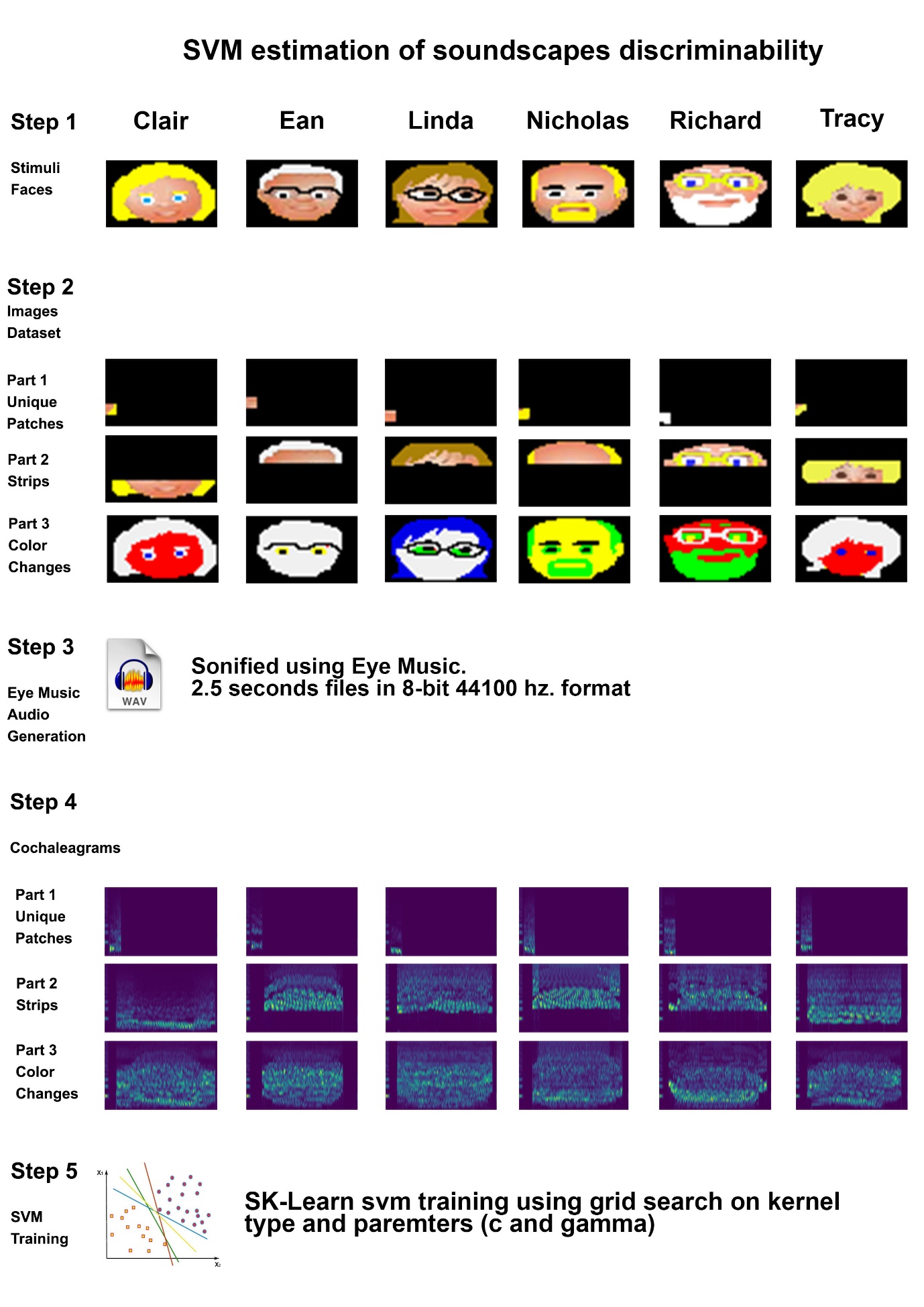


**Figure S1: Description of the SVM pipelin:** to test whether some unique characteristics of the face exemplars could be used to identify the face, potentially even without specific training, we trained an SVM model on cochleograms created from all 5X5 unique patches which were part of each face (part 1). After training a linear SVM on all cochleograms, the SVM correctly identified only one of the six faces, the equivalent of random chance level. To test whether the soundscapes themselves contained enough discriminable features, we used two different models: in part 2 we tested the model on horizontal “strips” similarly to those used in training; in part 3 we tested the model on cochleograms produced from soundscapes with color-changed features. The model was successful in correctly generalizing training to the full cochleograms representing the facesl. To create the cochleograms, an open-source Python library was used (pycochleagram: [GitHub - mcdermottLab/pycochleagram: Generate cochleagrams natively in Python. Ported from Josh McDermott's MATLAB code.](https://github.com/mcdermottLab/pycochleagram)).

**Supplemental movie:** **A congenitally blind adult identifies faces using auditory soundscapes:** despite never seeing faces, a congenitally blind adult can identify facial features and name specific characters using auditory information exclusively.

**Supplemental face soundscapes library**: All auditory face stimuli used in the presented experiments are available within this soundscapes library.

1. Sharan, R. V. & Moir, T. J. Subband Time-Frequency Image Texture Features for Robust Audio Surveillance. *IEEE Trans. Inf. Forensics Secur.* **10**, 2605–2615 (2015).

2. Gao, B., Woo, W. L. & Khor, L. C. Cochleagram-based audio pattern separation using two-dimensional non-negative matrix factorization with automatic sparsity adaptation. *J. Acoust. Soc. Am.* **135**, 1171 (2014).

3. Sharan, R. V. & Moir, T. J. Pseudo-color cochleagram image feature and sequential feature selection for robust acoustic event recognition. *Appl. Acoust.* **140**, 198–204 (2018).
